# Current status of treatment and disease burden of a cohort of hemophilia B in China

**DOI:** 10.3389/fpubh.2023.1303787

**Published:** 2024-01-24

**Authors:** Yiwen Huang, Chuchuan Wan, Tao Guan, Xiaoyu Xi

**Affiliations:** ^1^The Research Center of National Drug Policy & Ecosystem, China Pharmaceutical University, Nanjing, China; ^2^Beijing Hemophilia Home Care Center, Beijing, China

**Keywords:** China, hemophilia B, economic burden, cost, prophylaxis

## Abstract

**Objective:**

Hemophilia B is a rare X-chromosome linked hereditary bleeding disorder. Patients require lifelong treatment and it is costly, but there is a lack of research in China on the treatment and burden for this group. Our aim was to review the actual treatment pattern of hemophilia B patients in China, and describe the financial burden and other disease burden from the patient’s perspective.

**Methods:**

Using data collected by the Beijing Hemophilia Home Care Center, descriptive statistics were made on the sociodemographic characteristics and treatment of patients. The annual drug costs were calculated according to the actual factor dosage and price.

**Results:**

During the study period, 29.9% of the patients only received on-demand treatment, while the rest of the patients received varying numbers of prophylaxis treatment. The total cost of clotting factors for 341 patients in one year was 16.0 million CNY ($2.5 million), with 46990.8 CNY ($7283.7) per patient. The drug cost of prophylaxis was significantly higher than that of on-demand treatment. The amount of prothrombin complex concentrates used by patients was the largest, more than 5 times of recombinant coagulation factor IX. Based on the average annual wage and average working time of Chinese employees in 2021, the average annual wage loss of HB patients reached 31544.2 CNY ($4889.4). The results of the questionnaire showed that 77.1% and 65.3% of patients had chronic pain and acute pain of different frequencies.

**Conclusion:**

The level of prophylaxis for Chinese patients is low; safer and more effective recombinant drugs are not widely available. Patients also face a high burden of drug costs, as well as indirect costs that cannot be underestimated. Therefore, continued efforts are needed to improve the quality of life of patients by reducing their financial burden and promote standardized treatment.

## 1 Introduction

Hemophilia B (HB), a rare X-chromosome linked hereditary bleeding disorder (1), has been included in the Circular of Publishing the First List of Rare Diseases in China (2). HB usually occurs in males [global prevalence is 1 in 30,000 (3), and China’s is about 1 in 100,000 (4)], while it is extremely rare in females. Patients with HB have a deficiency of coagulation factor IX (FIX) activity due to mutations in the genes encoding FIX, which leads to coagulation dysfunction (5, 6). Bleeding is the most prominent clinical manifestation of HB, among which spontaneous bleeding, minor post-traumatic bleeding, and severe bleeding after wound or surgery are more common (7). As a lifelong disorder, hemophilia is associated with significant clinical burden driven by haemarthrosis, joint damage and pain, with subsequent negative impact on patients’ mental health, daily functioning and overall quality of life (8).

Given the nature of HB, replacement therapy of high-quality factor concentrates is essential (9). Patients with HB mainly receive clotting factor replacement therapy and surgical treatment for bleeding and joint problems. In addition, gene therapy which has shown promising results in some clinical studies (10). Replacement therapy is currently the primary and most effective clinical treatment for HB (11). It is used to replenish the FIX deficiency in patients and includes on-demand treatment and prophylaxis. The former is to address timely hemostasis after bleeding episodes, and the latter is a regular continuous replacement therapy to prevent bleeding (12). The *Chinese guidelines on the treatment of hemophilia (version 2020)* (5) and The *WFH Guidelines for the Management of Hemophilia, 3rd edition* (13) state that recombinant FIX (rFIX) or viral-inactivated prothrombin complex concentrates (PCCs) is the preferred alternative treatment for HB. Fresh frozen plasma (FFP) can be used if these clotting factor concentrates (CFCs) are not available. PCCs are the main product of HB replacement therapy in China today. However, they are not the most ideal products. Because PCCs contain coagulation factors II, VII, and X in addition to FIX components, some of which may lead to the risk of venous and arterial thrombosis or disseminated intravascular coagulation (DIC) formation with long-term high dose use.

The implementation of replacement therapy means that patients will be on medication for the rest of their lives, resulting in ongoing treatment costs, most notably for clotting factor medications. During the course of replacement therapy, some patients may develop inhibitors to clotting factor, which can reduce the effectiveness of replacement therapy. Additional costs and medication burden will be incurred if patients are treated with bypass agents [e.g., genetically recombinant activated coagulation Factor VII (rFVIIa) and immune tolerance induction (ITI) therapy]. Furthermore, incomplete treatment of long-term recurrent joint bleeding can damage the joint structure, resulting in joint deformity and loss of function. Worse still, joint replacement may be required, and patients have to endure pain during the recovery process while bearing the high cost of surgery. Studies have been conducted in Europe and the United States to demonstrate the clinical, human, and economic burden of severe HB patients (14, 15). However, most available studies (over 70%) on the burden of hemophilia in China did not distinguish between HA and HB or only targeted HA patients, and were conducted from a third-party perspective, analyzing direct treatment costs through health insurance system data. There is a lack of studies specifically focusing on patients with HB, where the sample size of hemophilia B patients is small or does not indicate the type of hemophilia (16, 17).

In view of the above, this study aims to review the actual treatment pattern of HB in China using data from the Beijing Hemophilia Home Care Center patient database, thereby aiming to identify the shortcomings in treatment. At the same time, the study also describes the financial burden and other disease burden from the patient’s perspective, so as to better understand the HB patients’ burden. These will help the relevant parties to explore better treatment strategies and burden reduction measures.

## 2 Method

### 2.1 Study design

The study was conducted using data from current patients (with a confirmed diagnosis of HB, excluding carriers) in the Beijing Hemophilia Home Care Center database. Sociodemographic information and treatment information in the database were mainly accessed. For the reason of data availability, usability, completeness, and quality, only patient data within one consecutive year from September 1, 2020 to August 31, 2021 were extracted. We also added a patient questionnaire study to investigate other patient burden, which consisted of two parts. One part was about health service utilization (visits within two weeks, hospitalizations within one year) and patient labor loss (work time lost due to illness, actual work time, and impact on productivity). The questions in this section were generated from literature reviews and expert interviews. The other part assessed pain levels using a visual analogue scale (VAS).

### 2.2 Data collection

The Beijing Hemophilia Home Care Center is the largest organization for hemophiliacs in China, with over 14,000 registered patients. The organization collects data on hemophiliacs on Chinese mainland through an application called “Hemophilia Home.” The App allows patients and their families to record information on bleeding, treatment, medical appointments, medication costs, etc.

Patient inclusion criteria: (1) be a citizen of the People’s Republic of China, regardless of age or sex; (2) knowingly and voluntarily register for the Hemophilia Home APP and agree to the APP registration agreement; (3) be diagnosed with hemophilia B by a regular hospital, excluding carriers. Patients register in the APP on their own, and their caregivers can register on behalf of infants, the elder or other people with mobility difficulties who cannot complete the registration on their own. Patients will record their use of each treatment in the APP for each episode, along with the cause, site, and symptoms of each bleeding. All other information will be registered only once.

In this study, we mainly used sociodemographic information and treatment data of patients in the database. Specifically, the sociodemographic included gender, age, education and employment status, marital status and health insurance information; the disease treatment information included disease severity and treatment records (including type of treatment, type and dosage of medication for each treatment). Additionally, a convenience sample of the active patient users was conducted in the form of an online questionnaire that patients voluntarily completed via the Internet, ultimately collecting data on patient health service utilization and loss to the labor force.

### 2.3 Statistical analysis

The raw database data were cleaned before analysis. The sociodemographic information of individual patients was corrected by verifying with the staff of the Beijing Hemophilia Home Care Center. We merged two types of medical insurance (i.e., urban resident basic medical insurance and new rural cooperative medical scheme) used in the APP into urban and rural resident basic medical insurance to reflect the current status in China. Also, patients with obvious logical errors in information were excluded.

The study could be divided into three parts: basic patient characteristics, treatment status, and patient burden. Only patients with relevant data were considered in the treatment and burden analysis. Basic patient characteristics included gender, age, marital status, education, and employment status. The treatment status includes disease severity, treatment modality, type of drug used and factor dosage. The financial burden of patients was reflected by direct costs. Only factor treatment costs were considered due to data limitations. Furthermore, since the database only counted patients’ drug use and did not record each drug cost in detail, it was necessary to use an appropriate formula to estimate the cost. The annual drug cost calculation formula is as follows. The conversion between the Chinese Yuan (CNY) and the US dollar was made using the average exchange rate of the CNY to the US dollar in 2021, 6.4515 (Number of people completely unemployed due to illness * average weekly working hours + number of people whose working hours are partially affected by illness * average weekly working hours affected) * average hourly wage * 52^#^.

*#The average weekly working hours and average wages are obtained from the public data of the National Bureau of Statistics of China.*

Data were presented as means with standard deviation (SD) or medians for continuous variables and as frequencies with percentages for categorical variables. Given that the distribution of drug cost data did not satisfy normality and chi-square, a nonparametric test, the Kruskal-Wallis H test, was used in this study to compare differences in factor doses and drug costs between patients with different treatment modalities or different levels of severity.

All statistical analyses were performed using SPSS 26, Microsoft^®^ Excel 2016 and Stata 15.

## 3 Results

### 3.1 Sociodemographic characteristics

A total of 1,654 patients’ sociodemographic information was collected, the majority of whom were male (99.8%, *n* = 1,651). The mean age of the patients was 22.4 (±15.1) years, with 53.7% (*n* = 889) and 46.3% (*n* = 765) of adult (≥18) and underage (<18) patients, respectively. In our sample, 54.2% (*n* = 411) of the patients were of marriage age (The legal marriage age in China is 22 for men and 20 for women.) were unmarried, 4.7% (*n* = 36) were divorced or widowed, and the rest were married. Considering the large age span of patients, the education and employment status of patients were combined and analyzed. Of the total patients, 163 (9.9%) patients were in the preschool stage; 769 (46.5%) patients were in the education stage, but 41.4% (*n* = 318) of them were out of school. Additionally, 519 (31.4%) patients were unemployed, and the rest worked normally or retired (12.2%, *n* = 203) (Table 1).

**Table 1:** Sociodemographic characteristics of HB patients.

Items (*N* = 1,654)	*n* (%)/mean (SD)
**Gender**	
Male	1,651 (99.8%)
Female	3 (0.2%)
**Age**	**22.4 (15.1)**
Minors (<18)	765 (46.3%)
Adults (≥18)	889 (53.7%)
Marital status	
Unmarried	411 (54.2%)
Married	312 (41.1%)
Others (divorced/widowed)	36 (4.7%)
**Education and employment status**	
Preschool	163 (9.9%)
Educated and in school	451 (27.3%)
Educated but out of school	318 (19.2%)
Employed	191 (11.5%)
Unemployed	519 (31.4%)
Retired	12 (0.7%)

SD, standard deviation.

### 3.2 Treatment

A total of 341 patients were included in the analysis of treatment. Patients were diagnosed as having mild (5 < IU/dl < 40), moderate (1 < IU/dl < 5), or severe (IU/dl < 1) hemophilia based on factor activity levels. The vast majority of the 341 patients had moderate and severe HB, 48.4% (*n* = 165) and 48.7% (*n* = 166), respectively, and only 10 (2.9%) had mild HB (Table 1).

Because the database recorded the type of each treatment during the study period, patient treatment types were classified into three categories: having had on-demand treatment only, prophylactic treatment only, or both. During the study period, 178 (52.2%) had received both on-demand and prophylactic treatment, 61 (17.9%) received only prophylaxis, and the remaining 102 (29.9%) received only on-demand therapy. In terms of different severity levels, the percentages of the three treatment modalities were basically the same. About half (52.7%) of moderate patients and half (50.0%) of severe patients administered a combination of on-demand therapy and prophylaxis. Relatively, the proportion varied more due to the small number of patients with mild disease. Over the study period, the total amount of FIX used was 7.7 $\times 10^{6}$ IU, with 22660.7 IU per patient. The mean factor dosage was higher in moderate and severe patients than in mild, but the Kruskal-Wallis H test did not show a significant difference (*p* > 0.05). The total amount of PCCs used was the highest, reaching $6.5\times 10^{6}$ IU, followed by rFIX ( $1.2\times 10^{6}$ IU), while human coagulation factor IX ( $1.3\times 10^{4}$ IU) was the least (Table 2).

**Table 2:** Treatment status.

Items	*n* (%)			
Severity		Only on-demand	Only prophylaxis	A combination of both
Mild	10 (2.9%)	2 (20.0%)	0	8 (80.0%)
Moderate	165 (48.4%)	46 (27.9%)	32 (19.4%)	87 (52.7%)
Severe	166 (48.7%)	54 (32.5%)	29 (17.5%)	83 (50.0%)
**Treatment modality**				
Only on-demand	102 (29.9%)			
Only prophylaxis	61 (17.9%)			
A combination of both	178 (52.2%)			
**clotting factor**				
Total quantity (IU)	7,727,300			
Per patient (IU/person)	22660.7			
Mild	8390.0		chi2 (2)=1.711 Prob = 0.4251	
Moderate	21160.0			
Severe	25012.0			
PCCs (IU)	6,522,300			
rFIX (IU)	1,192,000			
human coagulation factor IX (IU)	13,000			
**Average annual factor dosage** (IU/Kg)	619.5			
For on-demand therapy (IU/Kg)	194.3			
For prophylaxis (IU/Kg)	656.2			
Minors (<18 years)	845.8			
Adults (≥18 years)	318.9			
**Number of injections per year**				
Mean (±SD)	29 (±31.8)			
Range	(1–176)			

IU, international unit; PCCs, prothrombin complex concentrates; rFIX, recombinant factor IX; SD, standard deviation.

**Figure 1:**
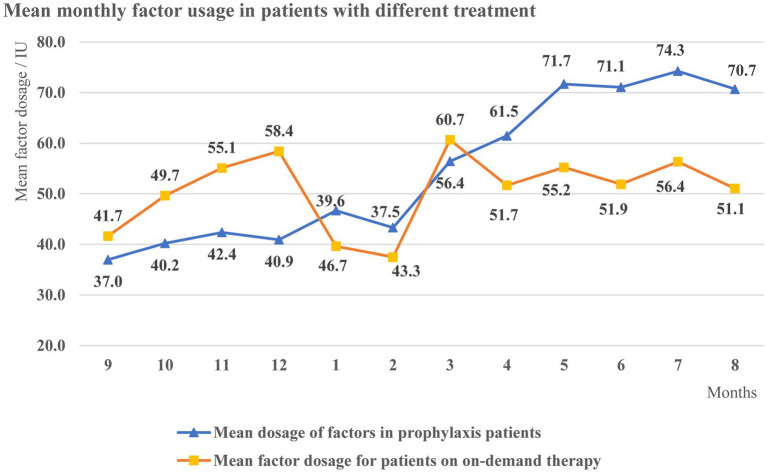
Mean monthly factor dosage in patients with different treatment.

In view of the treatment level, the average annual factor dosage of 341 patients was 619.5 IU/Kg, of which the average annual factor dosage was 656.2 IU/Kg in 239 patients with prophylaxis and 194.3 IU/Kg in 280 patients with on-demand treatment. The mean factor dosage of patients with prophylaxis showed a fluctuating upward trend (37.0–74.3 IU/Kg), while there was no significant trend in the mean dosage of on-demand treatment patients (Figure 1).

### 3.3 Economic burden

The total cost of clotting factors for 341 patients during the study period (total of one year) was 16.0 million CNY ($2.5 million) calculated according to the aforementioned method, with a mean of 46,990.8 CNY ($7283.7) per patient and a median of 15,262.5 CNY ($2365.7). Of these, the cost for prophylaxis amounted to 11.7 million CNY ($ 1.8 million), with an average cost of 48,962.2 CNY ($7589.3) per patient with prophylaxis (*n* = 239), and the cost for on-demand therapy mounted to 4.3 million CNY ($0.7 million), with an average cost of 15434.9 CNY ($2392.5) per on-demand patient (*n* = 280) (Table 3). As each treatment was recorded as either “prophylaxis” or “on-demand,” the number of people corresponding to the two treatments mentioned above (*n* = 519) was greater than the total number of patients (*n* = 341).

**Table 3:** Result of Kruskal-Wallis H.

	Obs	Rank sum	chi2 (2)	Prob
**Treatment**			72.858	0.0001
On-demand (1)	102	10822.00		
prophylaxis (2)	61	10042.50		
combination (3)	178	37446.50		
**Severity**			4.325	0.1151
Mild	10	1172.50		
Moderate	165	27475.50		
Severe	166	29663.00		

Groups	Rank means difference	Critical value	Prob
(1) & (2)	58.52	38.20	0.000123 (S)
(1) & (3)	104.26	29.31	0.000000 (S)
(2) & (3)	45.75	35.01	0.000880 (S)

Obs, the number of observations in the current dataset.

The results of the Kruskal-Wallis H test for drug costs between patients with different treatment modalities and between severity levels are shown in Table 3. There was a significant difference in drug cost between patients with the three treatment modalities (*p* < 0.05), while there was no significant difference among patients with different levels of severity.

### 3.4 Other disease burden

For the further questionnaire study, 153 valid questionnaires were obtained. In the two weeks prior to the questionnaire study, 52 (34.0%) had a visit (both outpatient and emergency) with an average of 1.9 visits in two weeks. This indicated that there was a 60% average visit rate of patient within a two-week period. Thirty-two patients (20.9%) had been hospitalized in the previous year, with an average of 1.9 hospitalizations per year (Table 4).

**Table 4:** Other disease burden.

items	*n* (%)/mean (SD)
Visits within two weeks	
No	101 (66.0%)
Yes	52 (34.0%)
Average number of visits (per fortnight)	1.9
Hospitalization within one year	
No	121 (79.1%)
Yes	32 (20.9%)
Average number of hospitalizations (per year)	1.9
Chronic pain frequency	
Never	35 (22.9%)
Occasionally	64 (41.8%)
Sometimes	35 (22.9%)
Often	15 (9.8%)
Always	4 (2.6%)
Chronic pain frequency	
No pain	1 (0.8%)
Mild	70 (59.3%)
Moderate	37 (31.4%)
Severe	10 (8.5%)
Acute pain frequency	
Never	53 (34.6%)
Occasionally	71 (46.4%)
Sometimes	23 (15%)
Often	6 (3.9%)
Always	0
Acute pain level	
No pain	0
Mild	37 (37.0%)
Moderate	37 (37.0%)
Severe	26 (26.0%)
Work status (*n* = 26)	
Work normally	7 (26.9%)
Work affected by HB	19 (73.1%)
Average time of work affected (hours/week)	30.2 (40.4)

Thirty-five of 153 (22.9%) reported never having chronic pain, the majority of patients (64.7%, *n* = 99) had chronic pain occasionally or sometimes, 90.7% (*n* = 107) of those with chronic pain had mild or moderate pain, and 8.5% (*n* = 10) had severe chronic pain. Regarding the frequency of acute pain, 53 (34.6%) patients had no acute pain and the majority of patients with occasional or sometimes acute pain (61.4%, *n* = 94). Again, mild to moderate pain was predominant in those with acute pain (74.0%, *n* = 74) (Table 4).

The results from the 153 responses to the questionnaire showed that the work time lost due to HB was 30.2 h per week. This numerical value was used to estimate the work time and wages lost due to illness for that patient overall (*n* = 1,654). The average annual wage in China in 2021 was 85,945 CNY ($13321.8), and the average weekly working time was 47.8 h, or an average wage of 34.6 CNY ($5.4) per hour, resulting in a lost wage of 52,170,000.4 CNY ($8,087,129.3) for 1,654 patients in one year, or 31,544.2 CNY ($4889.4) per patient.

Using the sum of patient’s drug costs and lost wages to represent the total patient costs, the total annual cost was 68,197,963.3 CNY ($10,570,869.3), or an average of 78,530.0 CNY ($12,173.1) per patient.

## 4 Discussion

This study provides an overview of the treatment and disease burden of 1,654 hemophilia B patients from across China based on the Beijing Hemophilia Home Care Center’s patient database. As few studies in China have focused on this special group of HB patients, this study analyzes their treatment and burden at the national level, which can fill the gap in knowledge on burden to a certain extent. Meanwhile, it objectively established that HB patients are mostly troubled by the disease in daily life; and they faced a high financial burden and need improved treatment level.

A total of 97.1% of patients in this study had moderate or severe disease, similar to existing studies [81.7% (18), 90.0% (19)]. Nevertheless, mild patients may have been overlooked because of the lack of obvious preexisting bleeding resulting in delayed diagnosis. Approximately 30% of patients received only on-demand treatment during the study, and not all of those receiving prophylaxis received standard prophylaxis on a regular basis, leaving a gap in treatment patterns. Sixty-four per cent of patients with prophylaxis were under 18 years of age. The average annual factor dosage among prophylaxis patients was 656.2 IU/Kg, including 845.8 IU/Kg in children (<18 years) and 318.9 IU/Kg in adults (≥18 years), which is lower than in a single-center study at the Tianjin Blood Center (1328.0 IU/kg for prophylaxis and 878.8 IU/Kg in adults from 2014 to 2018) (18), while our low-dose prophylaxis regimen (5) (hemophilia B FIX preparation 20 IU/Kg once a week) requires at least 1,000 IU/Kg per year, which also indicates that there are still a large number of patients with extremely low levels of prophylaxis nationwide. For presentation purposes, we calculated the overall average patient dose monthly. The upward trend of dosing during prophylaxis may be caused by implementation of more intense regimens, but due to limited data, it is uncertain to accurately infer whether patients’ prophylaxis levels have improved over time. The treatment was the same among patients with moderate and severe disease, which suggests that those with moderate disease had similar phenotypes to those with severe disease, and were therefore included in the Registry, hence in our study. However, another reason may be that only those with severe disease felt the need to register on the APP.

The per patient factor cost calculated in this study was 46,990.8 CNY ($7283.7), and the median of 15,262.5 CNY ($2365.7) may be more representative due to the skewed distribution of the data. In a study of medical costs for urban hemophilia patients in China from 2010 to 2016 (16), the cost of coagulation factors for HB patients was 7,128.0 CNY ($1,104.9), and some difference between them may be due to the study being from a third-party payer perspective, including only inpatients, and having a small sample size (*n* = 80). The total cost of factor IX drugs for only 341 patients amounted to 16,024,000 CNY ($2,483,763.5) a year. The reported prevalence of hemophilia B in Chinese mainland was about 0.5/100,000 (4), which would result in over 300 million CNY ($46.5 million) in factor IX drug costs given the large population base in China. The ANOVA showed no significant differences in factor drug costs between patients with different severity levels. However, in the study by Buckner et al. (20), the total annual medical costs of hemophilia B patients increased with severity. This difference possibly resulted from inconsistencies in the calculation of costs, differences in patient baseline (only adult patients in their study), and differences in severity classification (mild, moderate, moderate–severe, and severe in their study). Again, another likely explanation is the difference in the diagnosis (and inclusion into the studies) of patients with mild bleeding phenotype (in mild and moderate patients).

In addition to economic losses, the physical and psychological harm to patients may be more serious. The average number of injections per year was 29, with a maximum of 176. Frequent injections brought physical injury to patients. Patients suffer from pain due to bleeding. In our study, about 77.1% of the patients experience chronic pain on a daily basis, which is generally consistent with existing studies [85% (14), 81% (15)], suggesting the importance of improving nursing care to alleviate the pain of patients. Better overall management including early diagnosis, early and adequate treatment as well as the availability of multidisciplinary comprehensive care etc. are of great benefit to patients.

It is necessary to note that this study has certain limitations. Firstly, only 341 patients had complete treatment records in the database, which may make the analysis of treatment modality and factor dosage different from the actual situation. Similarly, there is a bias in estimating the total indirect cost (wage loss) of patients based on the results of 153 supplementary questionnaires. Although some studies believed that the direct medical cost of hemophilia B was almost entirely caused by factor therapy (99%) (15), some studies showed that the treatment service accounted for 1/3 of the total medical cost (21), and other medical expenses of patients (such as other drug costs, joint surgery costs, etc.) were also worth considering. Furthermore, the analysis of patient bleeding pattern and comparison of pain in children and adults was hindered due to data limitations.

## 5 Conclusion

This study demonstrates the treatment profile and burden of hemophilia B patients in China. The level of prophylaxis for Chinese patients is low; safer and more effective recombinant drugs are not widely available. Inadequate treatment causes overwhelming suffering. Patients also face a high burden of drug costs, as well as indirect costs that cannot be underestimated. Therefore, patients with HB need access to more preferential policies to improve their quality of life by reducing their financial burden and promoting better, standardized treatment.

## Data availability statement

The raw data supporting the conclusions of this article will be made available by the authors, without undue reservation.

## Ethics statement

Ethical review and approval was not required for the study on human participants in accordance with the local legislation and institutional requirements. Written informed consent from the participants was not required to participate in this study in accordance with the national legislation and the institutional requirements.

## Author contributions

YH: Writing – original draft. CW: Writing – original draft. TG: Writing – review & editing. XX: Writing – review & editing.
